# Normal-Tension Glaucoma Complicated by a Giant Internal Carotid-Ophthalmic Artery Aneurysm

**DOI:** 10.1155/2024/3878152

**Published:** 2024-05-10

**Authors:** Sudhat Ashok, Andrew Pilling, Peterkin Lee-Kwen, Lee R. Guterman, Asher Weiner

**Affiliations:** ^1^Jacobs School of Medicine and Biomedical Sciences, University at Buffalo/State University of New York (SUNY), 955 Main St., Buffalo, NY 14203, USA; ^2^Department of Ophthalmology, Ross Eye Institute, University at Buffalo/State University of New York (SUNY), 1176 Main St., Buffalo, NY 14209, USA; ^3^Department of Neurosciences, Buffalo Mercy Hospital Catholic Health System, 565 Abbott Rd., Buffalo, NY 14220, USA

## Abstract

*Purpose*. We describe a patient with normal tension glaucoma (NTG) of several years whose management was complicated by the presence of a giant internal carotid-ophthalmic artery aneurysm. *Observations*. A 72-year-old woman presented to our glaucoma clinic with accelerated deterioration of her vision in her left eye (OS) over a 1-month period. Her ophthalmic history was most notable for bilateral NTG diagnosed 3 years prior which had been treated with several laser trabeculoplasty OS and topical bimatoprost 0.01% eye drops in both eyes (OU). Upon evaluation, her visual acuity OS had worsened, and visual field (VF) testing showed extensive progressive losses temporally and pericentrally OS over a year with stable IOP measurements and no neurological complaints. Given her atypical NTG progression, she was referred for an urgent neurological evaluation which revealed an unruptured giant left internal carotid-ophthalmic aneurysm. Following the successful treatment of the aneurysm with platinum coils, she continued to demonstrate additional bilateral ophthalmic changes including further progression of VF loss and RNFL thinning OS > OD on follow-up. *Conclusion and Importance*. Overall, this report describes a unique complication in the management of a patient with chronic bilateral NTG in the form of a giant internal carotid-ophthalmic aneurysm. Moreover, it highlights the need for clinicians to maintain a degree of suspicion for compressive lesions of the optic nerve when presented with atypical progression of VFs and/or visual acuity loss in glaucomatous patients.

## 1. Introduction

Glaucoma is a characteristic progressive optic neuropathy that may be associated with elevated intraocular pressure (IOP) or with normal IOP, the latter referred to as normal tension glaucoma (NTG) [1]. Since IOP is normal in such patients, the diagnosis of NTG requires careful consideration of masquerading (glaucoma-like) optic neuropathies including neurological etiologies [2]. However, differentiating NTG from neurological causes of optic disc cupping and visual deficits remains a challenge. Neurological conditions such as ischemic, hereditary or demyelinating optic neuropathies, and intraorbital or intracranial masses with compressive effects on the optic nerve or chiasm can all present with disc cupping and visual deficits that can mimic NTG [3, 4]. Therefore, NTG diagnosis requires a thorough review of a patient's medical history and careful examination of all possible etiologies.

## 2. Case Report

A 72-year-old Caucasian woman presented to the glaucoma clinic with a gradual worsening of vision in the left side of her left eye (OS) over a 1-month period. Medical and ophthalmic history included asthma, childhood trauma with a “black eye” OS, bilateral cataract extraction 5 years prior, and bilateral NTG diagnosed 3 years prior and treated with several laser trabeculoplasty OS and topical bimatoprost 0.01% eye drops in both eyes (OU). Progression of peripapillary retinal nerve fiber layer (RNFL) thinning on optical coherence tomography (OCT) OS and bilateral increase in optic disc cupping was noted 4 months prior, and a visual field deterioration OS was noted 1 month prior. The best corrected visual acuity was 20/20 OU 8 months prior. The patient denied headaches or other neurological issues.

**Figure 1:**
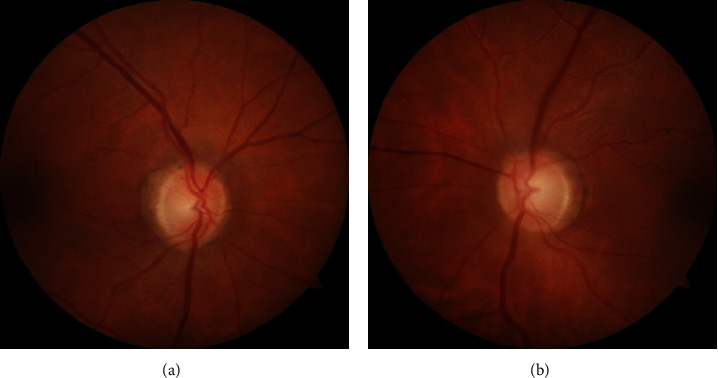
Photographs of right and left optic nerves at presentation, respectively. Cup/disc ratios: (a) 0.6 OD and (b) 0.75 OS.

**Figure 2:**
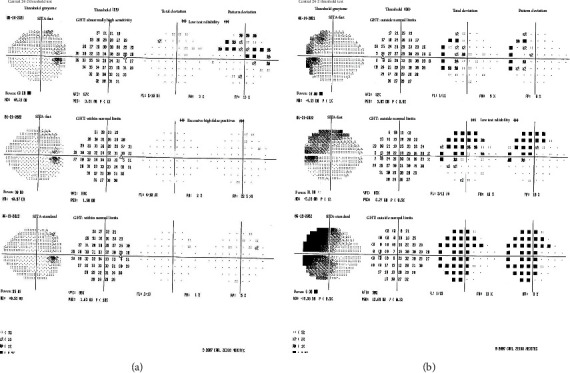
(a) Visual field testing of the right eye showing relative stability over a 12-month period. (b) Visual field testing of the left eye showing significant progressive temporal and paracentral loss over the same 12-month period.

On examination, the best corrected visual acuity was 20/20 OD but decreased to 20/50 OS with the patient noticing that the left side of each Snellen chart line was missing OS. There was no relative afferent pupillary defect (RAPD). IOP measured 8 mmHg OD and 6 mmHg OS by Goldmann applanation. Slit-lamp biomicroscopy revealed a stable cup-to-disc ratio of 0.6 OD but an increase to 0.75 OS in comparison to 4 months prior (Figure 1). Visual field (VF) findings were relatively stable OD (Figure 2(a)), but OS showed extensive superior more than inferior progressive loss temporally and pericentrally over a 12-month period (Figure 2(b)). In view of these significant changes, netarsudil 0.02% topical eye drops were added and the possible need for glaucoma surgery was discussed. However, since her visual deterioration OS was judged atypical of glaucoma, the patient was referred to an urgent neurological evaluation prior to surgery to rule out possible neurological etiologies for her deterioration OS.

**Figure 3:**
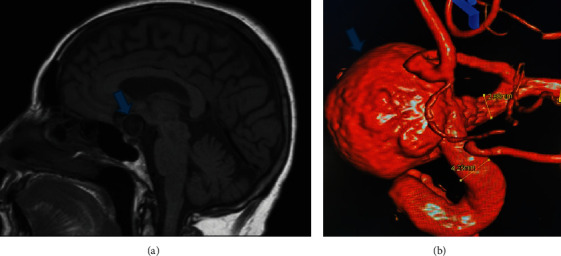
(a) MRI head without contrast showing a giant aneurysm (arrow) near the optic chiasm. (b) 3D CT angiogram confirming the presence of a giant aneurysm measuring 16 mm diameter with an 8 mm neck (arrow) arising from the internal carotid artery.

On neurological examination, a noncontrast MRI of the head revealed an unruptured giant left internal carotid aneurysm (Figure 3(a)). A 3D CT cerebral angiogram confirmed a 16 mm × 8 mm left carotid ophthalmic artery aneurysm with associated mass effect on the left optic nerve (Figure 3(b)). Upon neurosurgical consultation, the patient was found to have a visual loss that had progressed to light perception only in her left eye. The patient was taken for placement of a flow diverter stent into the left carotid adjacent to the aneurysm. However, this was unsuccessful secondary to the large defect in the left internal carotid artery (LICA), the aneurysm orifice. A balloon occlusion test with hypotensive challenge was performed to prepare the aneurysm and LICA to be occluded, followed by successful closure using detachable platinum coils. The patient recovered visual acuity of 20/50 OS following surgery and was subsequently discharged.

**Figure 4:**
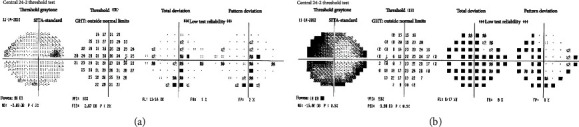
Visual field testing following the successful left internal carotid ophthalmic artery aneurysm coiling and occlusion. (a) Visual field testing of the right eye showing mild nonspecific worsening. (b) Visual field testing of the left eye showing further constriction of the field.

**Figure 5:**
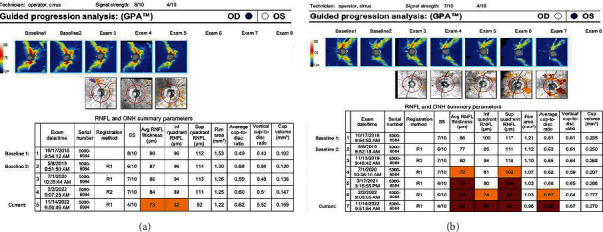
(a) RNFL and ONH summary parameters of the right eye demonstrating thinning inferiorly following neurosurgical intervention. (b) RNFL and ONH summary parameters of the left eye demonstrating progressive thinning of the RNFL despite neurosurgical intervention prior to the last reported RNFL measurement.

The patient was reevaluated at the glaucoma clinic 6 weeks later. RAPD was noted OS for the first time, and a repeat VF testing demonstrated mild nonspecific worsening OD and possible worsening constriction OS (Figure 4). OCT analysis showed further significant progressive thinning superiorly and inferiorly OS (Figure 5(b)). In addition, inferior RNFL thinning OD was noted for the first time (Figure 5(a)). IOP measurements remained stable.

## 3. Discussion

Cerebral arterial aneurysms occur in about 5% of the adult population, while internal carotid-ophthalmic artery aneurysms are rare and represent nearly 5% of cerebral arterial aneurysms [5, 6]. Visual symptoms relating to cerebral aneurysms include acute, gradual, or fluctuating visual loss due to compression of the optic nerve, by carotid or ophthalmic artery aneurysms [6]. Risk factors associated with internal carotid-ophthalmic artery aneurysms include female sex, hypertension, and smoking, the same as for all intracerebral aneurysms [7].

Prior to her presentation, our patient was being followed and treated for bilateral NTG for several years. She had a recent left VF deterioration over a 4-month period and a more acute left visual acuity loss with a noticeable left temporal paracentral defect over a 1-month period. This accelerated unilateral visual loss was judged atypical of glaucoma progression, and while the IOP-lowering medication regimen was increased and the possibility of glaucoma surgery was discussed, the patient was first referred for an urgent neurological evaluation which revealed a giant internal carotid-ophthalmic artery aneurysm compressing the left optic nerve.

Carotid-ophthalmic artery aneurysm is a rare cause of visual deficits and optic disc cupping that can mimic NTG [5]. Diagnosing neurological etiologies of visual deficits presenting as NTG is a challenge since routine neuroimaging as a screening tool in such eyes is not recommended [8]. Therefore, it is important for clinicians to assess a patient's complete history, clinical presentation, and progression to consider other potential causes of glaucoma-like optic neuropathies.

Our literature search revealed 3 case reports describing patients found to have an internal carotid-ophthalmic artery aneurysm with asymmetric cup-to-disc ratio [9–11]. Two of them were diagnosed with NTG [9, 11], and another with secondary high-tension glaucoma initially presenting with unilateral anterior uveitis, converting from negative to positive RAPD within several months [10], as did our patient. Other findings were dyschromatopsia in one case [9], unilateral visual field defects in two [10, 11], bilateral visual field defects in one [9], and unilateral optic disc pallor in two cases [9, 11]. One patient had a 2-year history of low vision in one eye with significant frontal headaches [11].

Comparatively, our patient is unique in several respects. She had a history of subjective unilateral visual loss of only 1-month duration with no previous similar episodes, no RAPD initially, and no headaches or other neurological symptoms. She was being treated and followed for bilateral NTG for several years prior to her recent symptomatic visual loss, providing a multiyear follow-up of OCTs and VFs. This allowed for the recognition of an acute change in the disease course with accelerated unilateral VF loss in addition to her more recent unilateral visual acuity loss, triggering a referral for an urgent neurological evaluation. Without such a longer-term follow-up, our patient might have presented with what appears to be advanced asymmetric NTG with a recent unilateral visual acuity loss that could have been attributed to her severe glaucoma not prompting a neurological workup.

On the other hand, having been followed and treated for chronic bilateral NTG added to the diagnostic challenge. Consideration of compressive lesions that may be responsible for progressive VF loss in addition to the already known NTG requires a high degree of suspicion. Clinicians must remember that while patients with NTG tend to have a paracentral defect on VF testing [5], it is commonly asymptomatic, and visual acuity is usually not affected early. Moreover, OCT RNFL usually reveals temporal and nasal thinning of the RNFL in compressive optic neuropathies (CON), whereas the inferior and superior RNFL are found to be more vulnerable in patients with glaucomatous optic neuropathy (GON) [12, 13].

However, other work suggests that the pattern of RNFL loss in CON is associated with the size and location of the compressive lesion and is not necessarily as clear-cut as described in literature [14]. For example, Qu et al. showed that the closer the compressive tumor was located to the internal opening of the optic canal in the perisellar location of their patients, the higher the likelihood of a glaucoma-like disc appearance [15]. The authors hypothesized that based on the size and location, the compressive lesion may obstruct the flow of cerebrospinal fluid (CSF) from the cranium into the orbit, potentially decreasing the retrolaminar CSF pressure and thereby making the optic head susceptible to glaucomatous changes. Similarly, several studies attribute the variation of VF damage observed in CON patients to the mass effect and/or ischemia damaging different portions of the optic chiasm [14, 15].

In this report, we observed significant progressive temporal and paracentral loss OS and nonspecific worsening OD on VF. OCT revealed progressive thinning superiorly and inferiorly OS and inferior RNFL thinning OD. Our patient's VF and OCT testing in combination with neuroimaging agree with these reports [14, 15], given the aneurysms perisellar location and the observed glaucomatous-like cupping, associated VF defect and RNFL thinning in the left eye. These findings highlight the challenge in interpreting the OCT RNFL and VF results of our patient and show that the findings expected in a pure compressive lesion, and in pure NTG, are blurred due to the presence of bilateral NTG in addition to the compressive lesion on the left optic nerve.

Lastly, our patient demonstrated additional bilateral ophthalmic changes following the successful treatment of her aneurysm, including further progression of VF loss and RNFL thinning L > R. These findings contrast with the three previously published case reports in which the patients were not observed to have worsening visual deficits bilaterally following the successful aneurysm closure [9–11]. We suspect that our findings may be the result of a combined effect of progressive bilateral NTG with continued vascular deprivation of the left optic nerve following her aneurysm closure.

## 4. Conclusion

This case underscores the need for clinicians to consider a neurological workup including neuroimaging in patients already treated for glaucoma when atypical progression of VFs and/or visual acuity loss are identified even in the absence of RAPD or any neurological symptoms to rule out compressive lesions of the optic pathways.

## Data Availability

The data used to support the findings of this study are included within the article.
